# Cysteines and Disulfide Bonds as Structure-Forming Units: Insights From Different Domains of Life and the Potential for Characterization by NMR

**DOI:** 10.3389/fchem.2020.00280

**Published:** 2020-04-23

**Authors:** Christoph Wiedemann, Amit Kumar, Andras Lang, Oliver Ohlenschläger

**Affiliations:** ^1^Institute of Biochemistry and Biotechnology, Martin Luther University Halle-Wittenberg, Halle, Germany; ^2^Leibniz Institute on Aging – Fritz Lipmann Institute, Jena, Germany

**Keywords:** disulfide bridge, cystine, protein, peptide, NMR, spectroscopy

## Abstract

Disulfide bridges establish a fundamental element in the molecular architecture of proteins and peptides which are involved e.g., in basic biological processes or acting as toxins. NMR spectroscopy is one method to characterize the structure of bioactive compounds including cystine-containing molecules. Although the disulfide bridge itself is invisible in NMR, constraints obtained via the neighboring NMR-active nuclei allow to define the underlying conformation and thereby to resolve their functional background. In this mini-review we present shortly the impact of cysteine and disulfide bonds in the proteasome from different domains of life and give a condensed overview of recent NMR applications for the characterization of disulfide-bond containing biomolecules including advantages and limitations of the different approaches.

## Introduction

Disulfide bridges formed between cysteine residues in peptides and proteins are fundamental building blocks for the molecular architecture and, thus, can govern basic biological processes. The formation of a disulfide bond by two side chain S^γ^ atoms of spatially proximal cysteines constitutes a two-electron oxidation process leading from reduced sulfhydryl groups of cysteines (S-H) to the oxidized cystine (S-S) residue. In cellular environments, this reaction is often supported and accelerated by enzymes like thioredoxin (Mahmood et al., 2013) or protein disulfide isomerases (Lee and Lee, 2017). Disulfide bridges can be formed intramolecular, in rarer cases even between two vicinal cysteines (Carugo et al., 2003), and constitute the only natural covalent link between polypeptides strands. In addition, they might occur as an intermolecular feature, sometimes leading to increased protein aggregation. Cleavage of disulfide bonds in biomolecules may result in the collapse of the native conformation and biological function. Thus, failures in formation or processing of disulfide bonds may lead to severe disorders by the accumulation of protein aggregates, by imposing cellular stress conditions and/or by leading to cell death (Rakhit and Chakrabartty, 2006; Hetz, 2012; Xu et al., 2014; Bechtel and Weerapana, 2017). Thus, nature has evolved a multitude of proteins with specialized biological functions based on molecular architectures involving different numbers of cystines. Only a few examples are Kunitz-type trypsin inhibitors (Otting et al., 1993; Cohen et al., 2019), multi-domain Kazal-type thrombin inhibitors like rhodniin (Van De Locht et al., 1995) and dipetalin (Schlott et al., 2002), growth factors (Christinger et al., 2004; Sitar et al., 2006), defensins (Szyk et al., 2006), neuropeptides like oxytocin (Bhaskaran et al., 1992) and vasopressin (Schmidt et al., 1991) or peptidic toxins (Elnahriry et al., 2019) and cyclotides (Park et al., 2017; De Veer et al., 2019; Huang et al., 2019).

Following the application of chiroptical techniques to uncover the structural features of a disulfide bond (Beychok, 1966; Van Wart et al., 1973; Menendez-Botet and Breslow, 1975), in the 1970s NMR spectroscopy started to emerge as method for structure determination of disulfide-bridged peptides and proteins (Ludescher and Schwyzer, 1971). Meanwhile, the power of this technique is highlighted, e.g., by the fact that 121/165 of 137/182 conotoxin structures deposited in the RCSB protein data bank or the ConoServer (Kaas et al., 2012), respectively, are NMR solution structures. NMR also allows to analyze structural and dynamic aspects of transient oxidative folding processes (Szekely et al., 2018) and to reveal conformational switching processes between disordered and folded states (Fraga et al., 2017) controlled by disulfide bridge formation. In the following sections, we present a short overview of NMR applications for the characterization of disulfide-bond containing biomolecules.

## Cysteine Abundance Analysis of the Proteome

To emphasize the special role of cysteines as a structure-forming or catalytic unit in the context of an evolutionary process, we present a short analysis of proteomes from different domains of life. Questions that arise are: (I) how many proteins of a proteome contain cysteines, (II) what is the average number of cysteines and disulfide bonds in a protein, (III) are there differences in the protein length or overall amino acid distribution among proteins with and without cysteines, and (IV) does the occurrence of cysteines correlate with the accumulation of other amino acids or amino acid patterns around these cysteines? In a first step, we selected different representatives from the three domains of life (Archaea: *T. gammatolerans*, Bacteria: *E. coli* and Eukaryota: *A. thaliana, D. melanogaster, S. cerevisiae, O. sativa, H. sapiens*) for which single defined proteome data sets are available in the UniProt database (UniProt Consortium, 2019). Except for *T. gammatolerans*, each selected data set is classified as a reference proteome in UniProt. Proteins in the data set are either annotated as reviewed (manually annotated) or unreviewed (full manual annotation still pending). Besides, we examined a data set that comprises all reviewed records in UniProt (referred herein as Reviewed SwissProt).

Eighty-three percent of all proteins annotated as reviewed in UniProt contain at least one cysteine and the number of cysteines accounts for 1.38% of all amino acids (a.a.) (Table 1, Figure S3). The median length of coding sequences of proteins for all reviewed entries in UniProt is 294 a.a. The cysteine-containing proteins are, on average, significantly longer (329 a.a.) compared to proteins that carry no cysteine (141 a.a.). On average 3 cysteines are present in proteins included in the SwissProt data set and 4 cysteines if only cysteine-containing proteins are considered.

**Table 1:** Proteomic analysis and disulfide bonds in reviewed proteins.

**Species (Uniprot proteome ID)**	**Number of proteins / proteins with Cys (percent)**	**Number of amino acids all / number of Cys (percent)**	**Median protein length all / proteins with Cys / proteins without Cys**	**Median Cys per protein all / proteins with Cys**	**Number of reviewed proteins / proteins with disulfide-bond (percent) / proteins with at least one interchain disulfide-bond**	**Median length of disulfide-bond containing proteins**	**Median disulfide-bonds (max.)**
Reviewed SwissProt	561 176 / 464 173 (83%)	201 585 439 / 2 787 012 (1.38%)	294 / 329 / 141	3 / 4	561 176 / 33 995 (6%) / 3 309	296	2 (166)
*A. thaliana* (UP000006548)	27 466 / 25 852 (94%)	11 122 644 / 207 856 (1.87%)	347 / 361 / 125	6 / 6	15 821 / 1 145 (7%) / 42	250	3 (8)
*D. melanogaster* (UP000000803)	13 798 / 13 018 (94%)	7 403 990 / 142 035 (1.92%)	395 / 412 / 150	7 / 7	3 559 / 349 (10%) / 30	513	3 (16)
*E. coli* (UP000000625)	4 391 / 3 694 (84%)	1 354 362 / 15 752 (1.16%)	271 / 296 / 137	3 / 3	4 389 / 98 (2%) / 7	284	1 (4)
*H. sapiens* (UP000005640)	20 660 / 19 979 (97%)	11 425 374 / 263 334 (2.30%)	410 / 421 / 125	9 / 9	20 305 / 3 591 (18%) / 334	362	2 (159)
*O. sativa* (UP000059680)	43 603 / 40 126 (92%)	13 382 401 / 260 236 (1.94%)	228 / 247 / 115	4 / 5	4 046 / 283 (7%) / 19	275	1 (16)
*S. cerevisiae* (UP000002311)	6 049 / 5 470 (90%)	2 936 363 / 37 272 (1.27%)	396 / 428 / 163	5 / 5	6 049 / 93 (2%) / 15	261	2 (14)
*T. gammatolerans* (UP000001488)	2 157 / 1 286 (60%)	636 517 / 3 603 (0.57%)	251 / 298 / 198	1 / 2	181 / 0 (0%) / 0	-	-

It is well-known that the median protein length in Eukaryotes is significantly longer than in Prokaryotes. Among Prokaryotes, Bacteria tend to have longer proteins, on average, than Archaea (Zhang, 2000; Skovgaard et al., 2001; Brocchieri and Karlin, 2005). Concerning the median protein length, the trends presented in Table 1 confirm the results observed by others (Zhang, 2000; Skovgaard et al., 2001; Brocchieri and Karlin, 2005) on a genomic level. With only a median protein length of 228 a.a. *O. sativa* significantly deviates from the average protein length of other eukaryotes. The genomic protein length distribution for each selected species is given in detail in Figure S5. Figures S7, S8 depict the genomic length distribution of cysteine-containing proteins and proteins without cysteines, respectively.

For a more realistic view of the median protein length and cysteine distribution in a cell/organism, the abundance weighted protein distribution is calculated and depicted (Table S1 and Figure S6). The protein abundance database [PAXdb, (Wang et al., 2015)], provides information about the whole genome protein abundance across different organisms and tissues. With the exceptions of *T. gammatolerans* and *S. cerevisiae* the abundance weighted median protein length is shorter compared with the genomic-based median protein length. Intriguingly, the abundance weighted median number of cysteines per protein is 4 to 5 in all selected eukaryotes and is lower than on the genetic level.

The frequency of cysteines seems to increase during evolution. While in *T. gammatolerans* only 60% of all proteins contain at least one cysteine, in eukaryotic proteomes, 92–97% of all proteins are cysteine-containing. This observation is also reflected in the species-specific cysteine percentage proportion of all amino acids (0.57% for *T. gammatolerans* and 2.30% for *H. sapiens*, Table 1 and Figure S3). Moreover, the median number of cysteines per protein tends to increase during evolution and reaches with 9 cysteines per protein in humans a maximum. For a detailed analysis of the genomic and abundance weighted cysteine distribution see Figures S9, S10, respectively. In the reviewed SwissProt data set the SCO-spondin proteins contain the highest number of cysteins [e.g., *G. gallus*: 584 cysteins (UniProtKB^1^: Q2PC93), *H. sapiens*: 563 cysteines (A2VEC9)]. It has to be noted that among the selected organisms the reference proteome of *D. melanogaster* includes a protein with 2647 cysteines (Dumpy, isoform Q; M9PB30). In contrast, the highest density of cysteines is observed in relatively short proteins/peptides. For example, conotoxins (P85019 or P0DPL4) and thiozillins (P0C8P6, P0C8P7) reveal with 46 and 43%, respectively, the highest content of cysteines. The “Small cysteine and glycine repeat-containing proteins” (e.g., A0A286YF46) and the “Keratin-associated proteins” (e.g., Q9BYQ5) show with ~40% the highest cysteine content in *H. sapiens*. If the difference in the amino acid distribution of non-cysteine-containing proteins compared to cysteine-containing proteins is considered (Figure S4), it is notable that, except for *T. gammatolerans*, in all data sets the leucine content is decreased, and at least one basic amino acid (lysine or arginine) content is increased, respectively. It is still subject to speculation if the structural or functional role of cysteines is compensated by an increase of, e.g., basic side-chain amino acids in non-cysteine-containing proteins.

In Figures S1, S2 we present the position-dependent amino acid frequency in cysteine-containing proteins. In each protein, which carries a cysteine, the amino acid distribution at each position N- and C-terminal stepwise next to cysteine is determined and compared to the overall amino acid distribution. The normalization is achieved by calculating the distribution ratio (amino acid distribution at position *n*/overall amino acid distribution). A normalized occurrence (distribution ratio) >1 implies a higher amino acid frequency at this position than expected from the overall distribution. The reverse is valid for a distribution ratio <1. It becomes clear that besides cysteine, mainly aromatic amino acids are more frequent around cysteines in all selected data sets. Particularly in the *H. sapiens* proteome the amino acids phenylalanine, histidine, and tyrosine reveal a more frequent pattern around cysteines than expected. These findings may reflect the widespread zinc finger structural motif.

Disulfide bonds are a central structural element which stabilizes the mature proteins' 3D structure and/or exhibit physiologically relevant redox activity (Bosnjak et al., 2014). They are mostly found in secretory proteins and extracellular domains of membrane proteins. Table 1 and Figures S11, S12 compile some statistical information about reviewed proteins with disulfide bonds. In the reviewed SwissProt data set, 6% of all proteins contain at least one disulfide bridge, and the median number of disulfide bonds is 2. As already mentioned above, for the content of cysteines, the conotoxins (e.g., P0DL39, P50983) also show with ~20% the highest content of disulfide bonds for all reviewed UniProt entries.

For the selected data sets, the content of proteins with at least one intra-chain disulfide bond increase during evolution (Table 1). Eighteen percent of all reviewed human proteins bear at least one disulfide bond. The maximal number of cystins/disulfide bonds currently observed in human proteins is 159 (Prolow-density lipoprotein receptor-related protein 1; 4544 a.a. in its canonical form; Q07954). However, as this protein contains 331 cysteines, it immediately becomes clear that not all of them under the same physical conditions form intramolecular disulfide pairs. When normalized by length, the shorter WAP four-disulfide core domain protein 3 (Q8IUB2) with 231 a.a. and 16 disulfide bonds takes over the pole position with ~7 bridges per 100 amino acids. In contrast, in *T. gammatolerans* no disulfide bonds are known for the reviewed proteins. The observation that the cysteine content in proteins increases during evolution can't be transferred clearly to the median number of disulfide bonds. In *H. sapiens* the median number of disulfide bonds is 2, whereas in *S. cerevisiae* it is also 2, but for *D. melanogaster* it is 3.

## NMR Spectroscopy & Prediction Techniques

**Figure 1:**
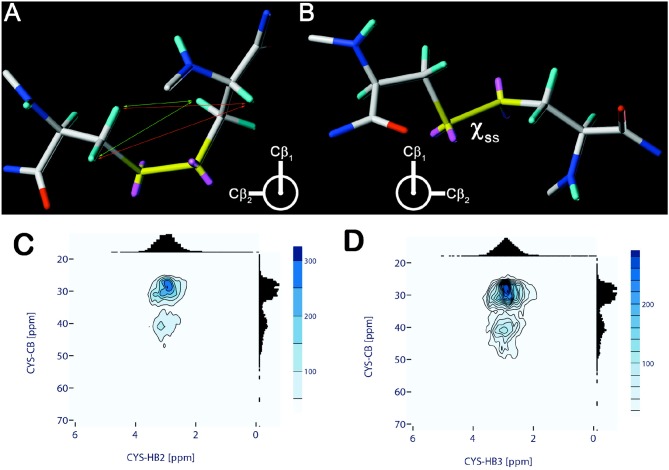
Chiralities of the disulfide bridge with χ_S−S_ torsional angle values of −90° **(A)** and +90° **(B)**. Coloring: H—cyan, C—gray, O—red, N—blue, S—yellow, free electron pairs—magenta. In **(A)** distance relations leading to typical cross peaks in NOESY-type spectra are marked (small arrows). Distance relations originating either from the H^α^ (orange) or the H^β^ protons (green) are indicated only for one of the two cysteines. **(C,D)** Chemical shift correlation of cysteine C^β^ and H^β2^ **(C)**, H^β3^ **(D)**, respectively. Chemical shift data and correlations are obtained and visualized from the Biological Magnetic Resonance Data Bank (BMRB) using a modified PyBMRB python module. Distribution values which are outside 10 times the standard deviation were removed from each correlation data set. Contour levels reflect the total number of correlations within.

Structurally, the disulfide linkage in a cystine displays a typical bond length of ~2.04 Å (Chaney and Steinrauf, 1974). The chirality of the disulfide linkage is a stereo-electronic consequence of the four free electron pairs on the two sulfur atoms. These electron pairs interact by repulsive forces with the neighboring β-carbon-containing groups, basically allowing two energetically favorable, mirror-imaged, and equally populated conformations for the C${}_{1}^{\text{β}}$-S${}_{1}^{\text{γ}}$-S${}_{2}^{\text{γ}}$-C${}_{2}^{\text{β}}$ torsion angle (χ_S−S_; Figures 1A,B) (Panijpan, 1977; Thornton, 1981). A newer study of 1,505 native disulfide bonds reported the average values of the χ_S−S_ torsion to be around−87° (left-handed) and +97° (right-handed) (Craig and Dombkowski, 2013). These torsion angle values are rather exceptional when compared to the other naturally occurring amino acids in peptides as those populate mainly side-chain torsions in the trans/anti (180°) or gauche (±60°) conformational range. In contrast to the redox state, no reliable prediction of the χ_S−S_ torsion angle from chemical shifts is available. However, the web-based approach “Disulfide by Design 2.0” (DbD2) (Craig and Dombkowski, 2013) allowed to correctly predict 96% of the disulfide chiralities based on an energy function reflecting the geometric characteristics found in an analysis of disulfide bonds in the PDB. Armstrong et al. (2018) recently reported about a prediction algorithm (DISH) for the two cysteine side-chain torsion angles χ_2_ and χ_1_ using a support vector machine. This approach had an overall accuracy of 81% for simultaneous prediction of both torsions and allowed to considerably reduce the spread in the protein backbone conformations in subsequent structure calculations.

With the advent of heteronuclear NMR techniques, analyses of the ^13^C chemical shift values of oxidized (S-S) or reduced (S-H) cysteines became available. Based on the ^13^C^α^ and ^13^C^β^ chemical shift data it could be deduced that the redox state is reflected in a distinct chemical shift pattern leading to two mainly non-overlapping areas for C^β^-shifts. These findings allowed the authors to suggest the following basic rule: “If the C^β^ shift is <32.0 ppm or >35.0 ppm, the redox state is assigned to reduced or oxidized, respectively” (Sharma and Rajarathnam, 2000). This empirical analysis was later supported by results of quantum chemical calculations of cysteine chemical shifts (Martin et al., 2010), which also rendered the ^13^C^α^ chemical shift value insensitive for an assignment of the redox state. As introduced above, disulfide bridges favor two distinct chiralities. Figures 1C,D shows the chemical shift distributions of the C^β^ vs. H^β2/3^ protons and Figure S13 for the other NMR active cysteine nuclei based on the actual BMRB data. It indicates that the C^β^/C^α^ distribution can be a supportive information for revealing the cysteines' redox state.

In addition to a pure NOE-based NMR structure determination, the measurement of residual dipolar couplings (RDC) allows to improve the resolution of 3D structures in case isotopically labeled compounds are available. Recently, the spider venom disulfide-rich peptide Ta1a was refined to a resolution of ~1.5 Å applying this approach (Ramanujam et al., 2020).

Lately, combining seleno-cysteine scanning and NMR analysis was shown to be a reliable approach for mapping disulfide bonds in cysteine-rich peptides and proteins (Denisov et al., 2019). The structurally conservative selenium substitution causes selective chemical shift changes of cysteine carbons involved in the mixed S–Se bond allowing identification by visual comparison of [^1^H,^13^C]-HSQC spectra of native and Sec-mutants.

## Conotoxins & Granulins

Conotoxins, small disulfide bridge-containing peptides found in marine cone snails, have attracted considerable scientific interest as they bind to ion channels. The pharmacological potential to modulate or block the ion channel activity and their synthetic availability make conotoxins promising candidates for new analgesics. However, Heimer et al. recently showed on the example of μ-PIIIA (three disulfide bonds) the complexity of the synthesis, purification, and analytical characterization of one specific isomer in the multitude of different potentially formed disulfide-bridged isomers of those cysteine-rich peptides (Heimer et al., 2018). With respect to this, ionic liquids have proven to be a promising solvent for controlling the oxidative folding process (Miloslavina et al., 2010).

The impact of deletion of disulfide bonds on the activity of α-conotoxins (two disulfide bonds) at human neuronal nicotinic acetylcholine receptors was studied employing NMR, Molecular Dynamics simulations and voltage-clamp techniques (Tabassum et al., 2017). The data supports the notion that the two disulfide bonds have been selectively conserved to create and stabilize a structural scaffold optimized for receptor binding.

Two recent publications presented structural relatedness between conotoxin structures and the granulin module, which was also solved by NMR and typically contains six disulfide bridges (Hrabal et al., 1996). For conotoxin Φ-MiXXVIIA a novel cysteine framework mimicking granulin and displaying anti-apoptotic activity was observed (Jin et al., 2017). Also for the conotoxin N_ext_H-Vc7.2 with three disulfide bridges the NMR structure determination revealed a granulin-like fold arising from the common inhibitor cystine knot framework (Nielsen et al., 2019). Based on further occurrences of this motif, e.g., in αD-GeXXa conotoxin, the authors conclude that the fold comprising two short, stacked β-hairpins stabilized by two parallel disulfide bonds might be an autonomous folding unit.

## Kazal-, Kunitz-, and Defensin-Type Folds

From earlier studies it is known that protease inhibitors, e.g., the thrombin inhibitors rhodniin (Van De Locht et al., 1995) and dipetalin (Icke et al., 2002), are composed of (repetitive) Kazal-type domains structurally shaped by three disulfide bridges. Recently, the NMR structure of CmPI-II, an inhibitor of trypsin, human neutrophil elastase, and subtilisin A, was elucidated and the complex with the latter modeled (Cabrera-Munoz et al., 2019). Similar to the recent structural study on SPINK6 (Jung et al., 2016) from the serine protease inhibitors of Kazal-type family (SPINK) (Feng et al., 2012), the authors describe a flexible N-terminal region and attribute the P2 site potential for alternative interactions in the complex formation.

Kunitz-type proteins, with bovine basic pancreatic trypsin inhibitor (BPTI) as the most extensively studied member (Berndt et al., 1992), display a compact conformation stabilized by three strongly conserved intra-chain disulfide bonds. Recently, (Banijamali et al., 2019) presented an NMR characterization of *Pseudocerastes persicus* trypsin inhibitor (PPTI) sharing structural similarities with dendrotoxins. By successive modeling, they could show that PPTI might block Kv1.1 potassium channels with the same mechanism as dendrotoxins. Also, Ixolaris, a potent tick salivary anticoagulant binding the coagulation factor Xa and the zymogen FX, shows a canonical Kunitz 3D structure (De Paula et al., 2019). However, the NMR and modeling results indicate that it exhibits a non-canonical inhibition interaction outside the active site of FX.

The pacifastin family of serine protease inhibitors found in animals and plants comprises short proteins exhibiting three β-strands which are again stabilized by three disulfide bridges (Simonet et al., 2002; Gaspari et al., 2004). These structural features can induce a stable, compact core and an extended binding loop.

Another peptide class displaying three disulfide linkages are defensins. Depending on the spacing of the cysteines and their pairing, three subfamilies (α, β, θ) are defined. Molecules of these classes share a similar structural fold (Lehrer and Lu, 2012; Dias Rde and Franco, 2015) and are facing interest as promising alternatives to conventional antibiotics. Recently, the NMR solution structure of rattusin expanded the structural repertoire of defensins by a scaffold formed by intermolecular disulfide exchanges between dimer units (Min et al., 2017).

## Kinases and Phosphatases

The C-terminal Src kinase (Csk) is a member of the CSK family of protein tyrosine kinases, which contains an SH2 domain carrying a unique disulfide bond which regulates the Csk kinase activity (Mills et al., 2007). The kinase activity of Csk was found to be strongly reduced upon the SH2 disulfide bond formation. Liu and Cowburn (2016) observed from X-ray data that only minor structural changes in the SH2 domain resulted from the disulfide bond formation. However, NMR measurements indicated that the reduced SH2 could bind slightly more efficiently with a Csk-binding protein-phosphorylated peptide.

Fms-like tyrosine kinase 3 is a member of the PDGFR (class III RTK) family containing disulfide bridges as well as free cysteines. By serine replacement of cytoplasmic cysteines evidence was found that oxidative modification of cysteine residues, e.g., by exogenous ROS, regulates the kinase activity of this clinically important oncoprotein (Bohmer et al., 2019).

For the closely-related members of the KIM-family protein-tyrosine phosphatases (PTP) (Machado et al., 2017) found significant differences in oxidation profiles coming along with different stabilization mechanisms. Whereas, striatal-enriched PTP and PTP-receptor type R stabilize their reversibly oxidized state by forming an intramolecular disulfide bond, in hematopoietic PTP the unexpected formation of a reversible intermolecular disulfide bond was observed.

## Concluding Remarks

The cited examples illustrate that cysteine disulfide bridging is an essential and highly evolved natural feature for the stabilization of peptide and protein structures and for modulation of biological activities. This finding is underlined by the extraordinary distributions of cysteines found in the proteomic data of different species/kingdoms. Current NMR and X-ray techniques allow defining the molecular structures of disulfide-rich biomolecules in high resolution. As disulfide bridges constitute the only natural covalent link between polypeptides strands, the acquired knowledge on their contribution to molecular scaffolding supports engineering of new cystine-based compounds with new functional (Nagarajan et al., 2018) or dynamical features (Gutmans et al., 2019), enhanced stability (Dombkowski et al., 2014), ultimately, aiming at improved pharmaco-kinetic and -dynamic properties for new therapies and treatment approaches. However, disulfide bonds tend to be unstable under reducing conditions, i.e., in many physiological situations, which triggered search for therapeutic compounds to make use of chemical modifications to stably replace these bonds. Thus, stable, non-reducible dicarba-bridged analogs were reported e.g., for oxytocin (Stymiest et al., 2003), for α-conotoxins of subtypes α-ImI, Vc1.1 and RgIA (MacRaild et al., 2009; Van Lierop et al., 2013; Chhabra et al., 2014) or, recently, insulin (Van Lierop et al., 2017).

## Author Contributions

CW, AK, AL, and OO equally contributed to the preparation of the manuscript. OO approved the final version.

## Conflict of Interest

The authors declare that the research was conducted in the absence of any commercial or financial relationships that could be construed as a potential conflict of interest.

## Footnotes

^1^In the following text the UniProtKB codes are referenced in brackets.

## Abbreviations

NMR: nuclear magnetic resonance
a. a.: amino acids.
