# Supplementary material for: Cysteines and Disulfide Bonds as Structure-Forming Units: Insights From Different Domains of Life and the Potential for Characterization by NMR
Source: Front Chem. 2020 Apr 23;8:280. doi: 10.3389/fchem.2020.00280 (PMC7191308; doi:10.3389/fchem.2020.00280)
Supplement: Supplementary file 1 [file Data_Sheet_1.pdf]

Supplementary Material

Cysteines and disulfide bonds as structure-forming units: insights from different domains of life and the potential for characterisation by NMR

Christoph Wiedemann[1*], Amit Kumar[2], Andras Lang[2], Oliver Ohlenschläger[2*]

[1] Institute of Biochemistry and Biotechnology, Martin Luther University Halle-Wittenberg, Germany

[2] Leibniz Institute on Aging – Fritz Lipmann Institute, D-07745 Jena, Germany

*** Correspondence:**
Christoph Wiedemann
christoph.wiedemann@biochemtech.uni-halle.de
Oliver Ohlenschläger
oliver.ohlenschlaeger@leibniz-fli.de

13 Figures and 1 Table

Supplementary Material

1 Supplementary Figures

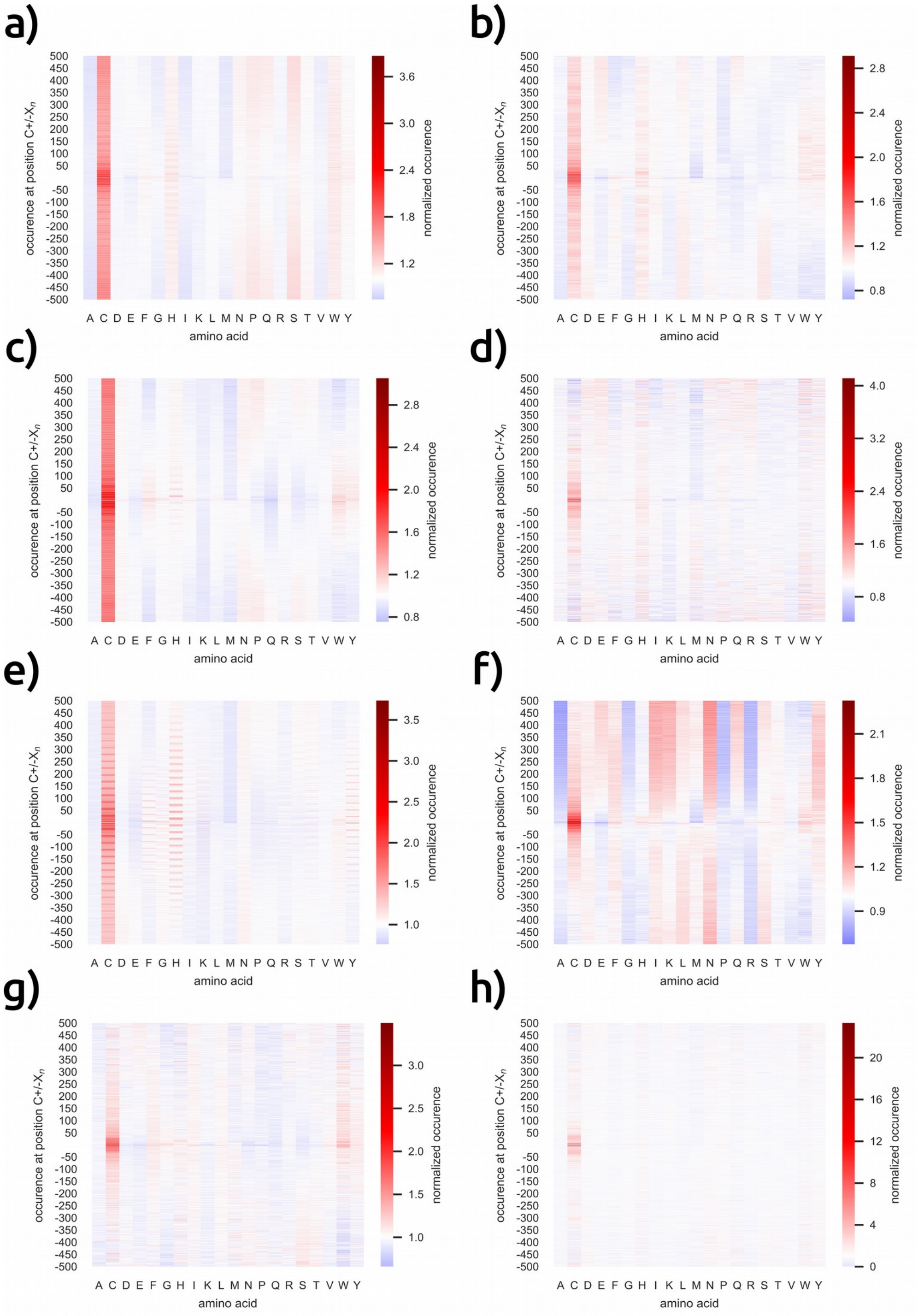

Figure S*1* The normalized amino acids occurrence N- (Cys-Xn) and C-terminal (Cys+Xn) next to a cysteine for the reviewed SwissProt (A), *A. thaliana* (B), *D. melanogaster* (C), *E. coli* (D), *H. sapiens* (E), *O. sativa* (F), *S. cerevisiae* (G) and *T. gammatolerans* (H) data set. The position of a cysteine is defined as 0. The normalization is achieved by calculating the distribution ratio (amino acid distribution at position n/overall amino acid distribution). A normalized occurrence (distribution ratio) >1 implies a higher amino acid frequency at this position than expected from the overall distribution and is color-coded in red. The reverse is true for a distribution ratio <1 and is color-coded in blue.

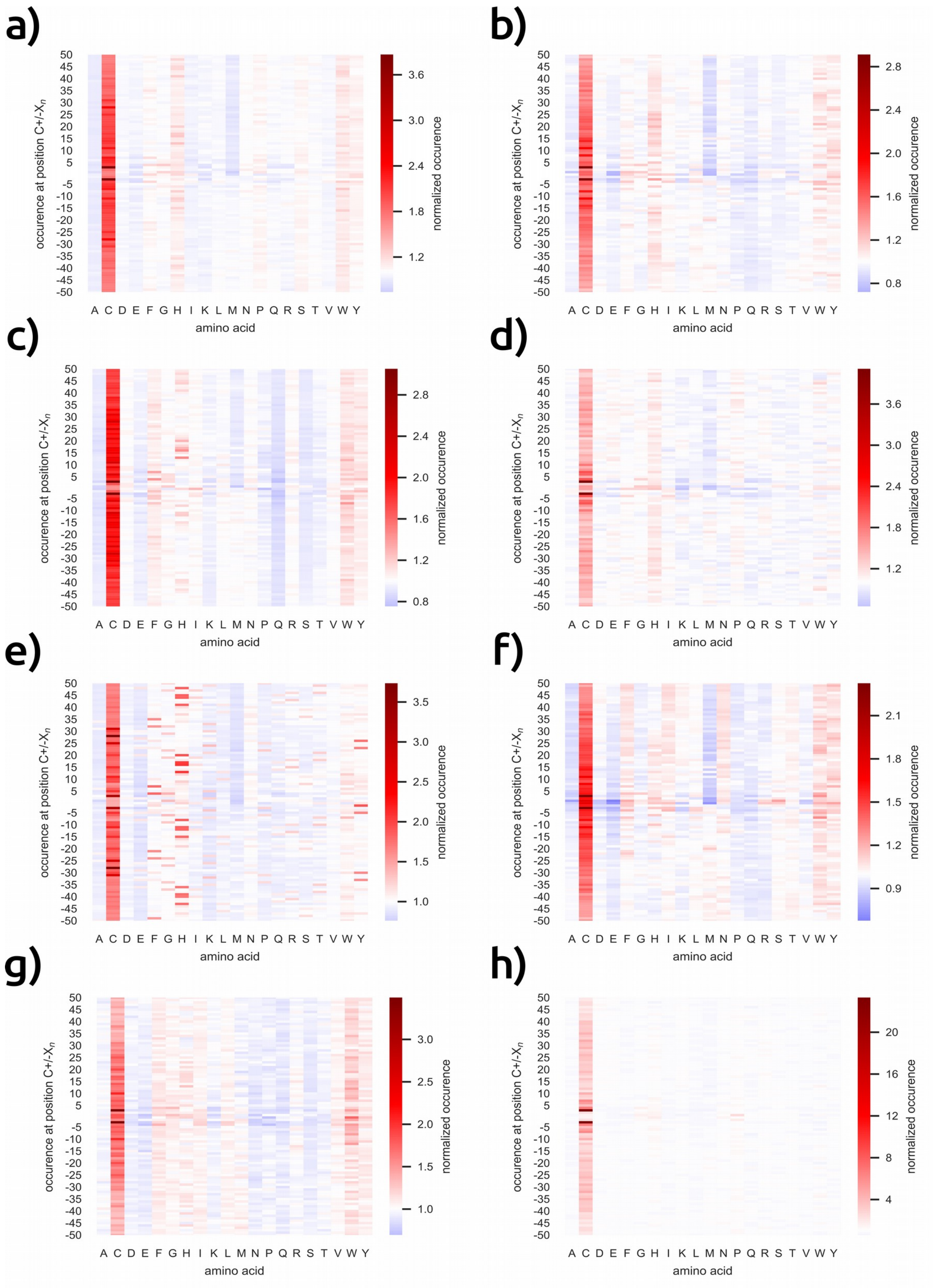

Figure S2 The normalized amino acids occurrence N- (Cys-X_n) and C-terminal (Cys+X_n) next to a cysteine for the SwissProt (a), *A. thaliana* (b), *D. melanogaster* (c), *E. coli* (d), *H. sapiens* (e), *O. sativa* (f), *S. cerevisiae* (g) and *T. gammatolerans* (h) data set. The position of a cysteine is defined as 0.

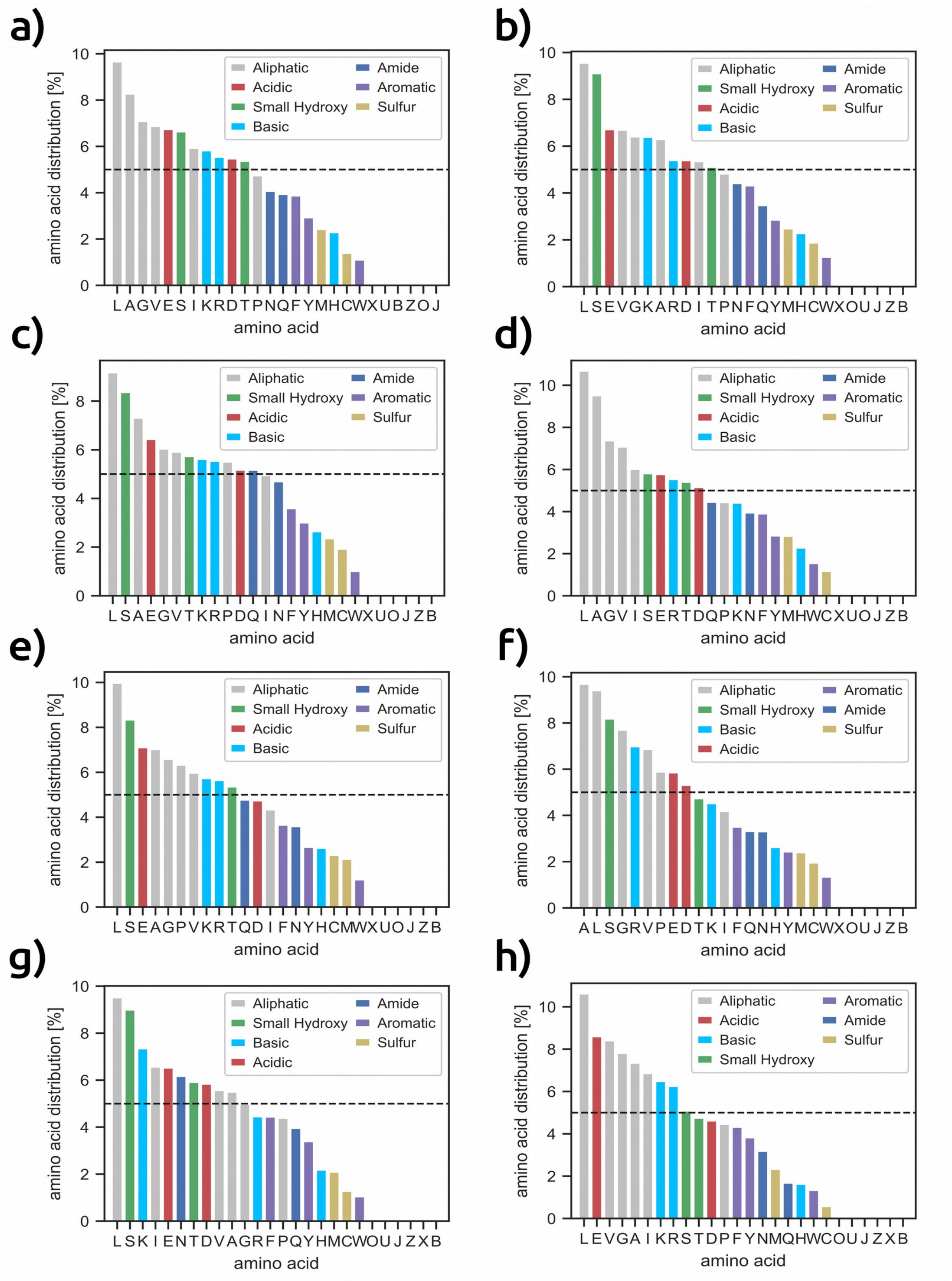

Figure S3 Amino acid distribution for the SwissProt (a), *A. thaliana* (b), *D. melanogaster* (c), *E. coli* (d), *H. sapiens* (e), *O. sativa* (f), *S. cerevisiae* (g) and *T. gammatolerans* (h) proteome deposited in UniProt. Expanded IUPAC single letter amino acid code is applied.

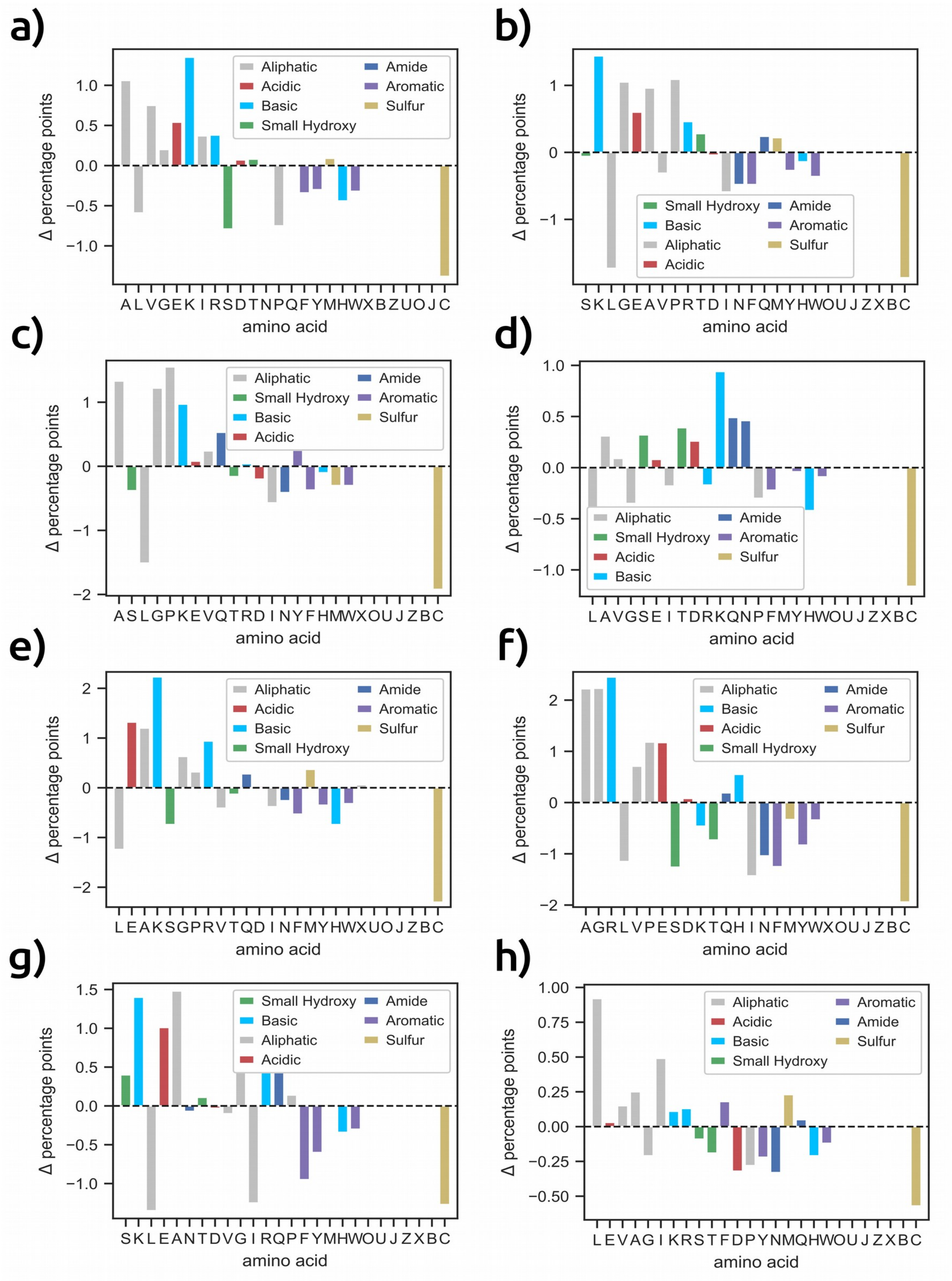

Figure S*4* Difference in amino acid distribution between cysteine-free and cystein-containing proteins for the SwissProt (a), *A. thaliana* (b), *D. melanogaster* (c), *E. coli* (d), *H. sapiens* (e), *O. sativa* (f), *S. cerevisiae* (g) and *T. gammatolerans* (h) proteome deposited in UniProt. Expanded IUPAC single letter amino acid code is applied.

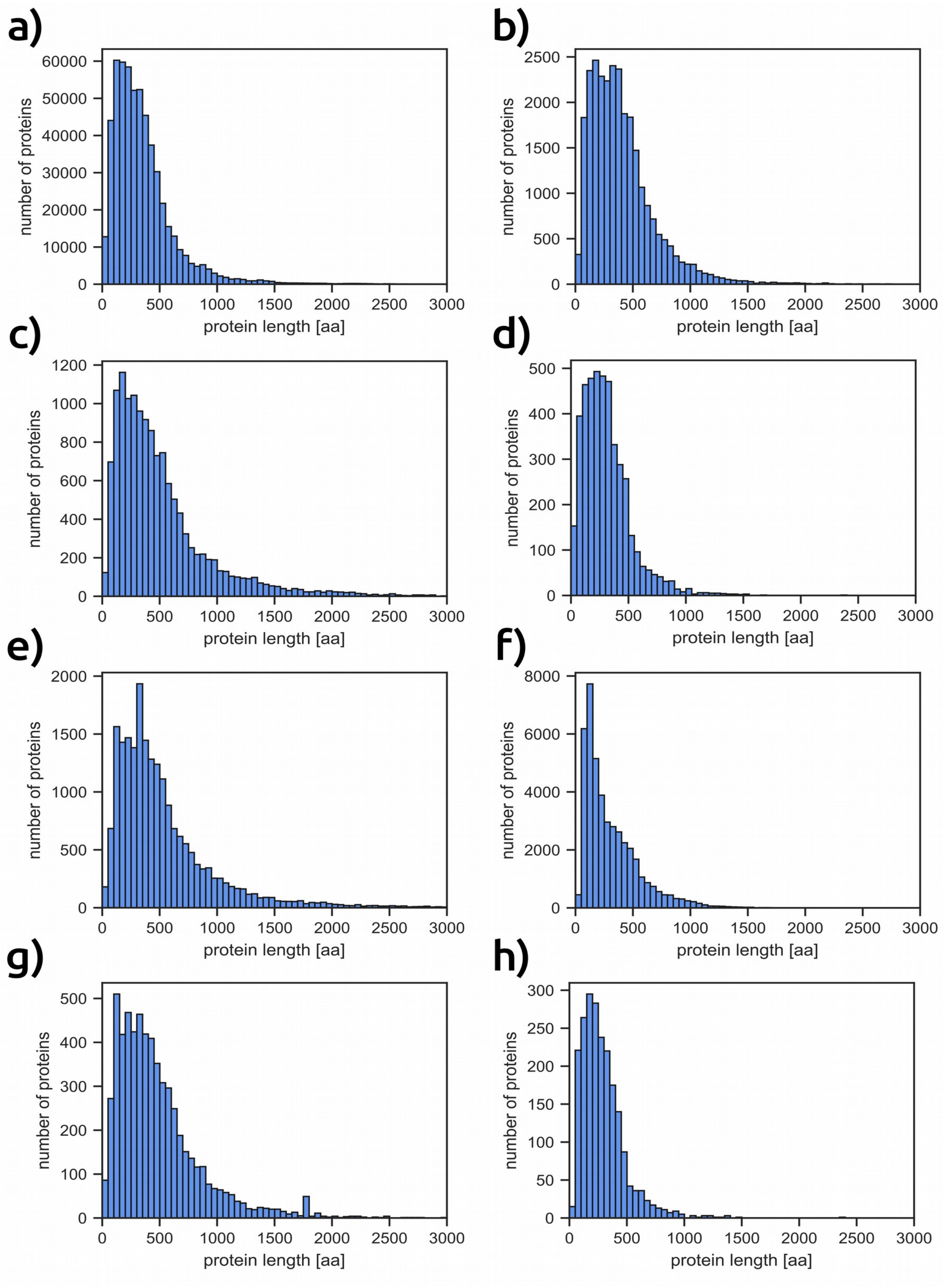

Figure S5 Genomic protein length distribution for the SwissProt (a), *A. thaliana* (b), *D. melanogaster* (c), *E. coli* (d), *H. sapiens* (e), *O. sativa* (f), *S. cerevisiae* (g) and *T. gammatolerans* (h) proteome deposited in UniProt. Bin size is 100 a.a.

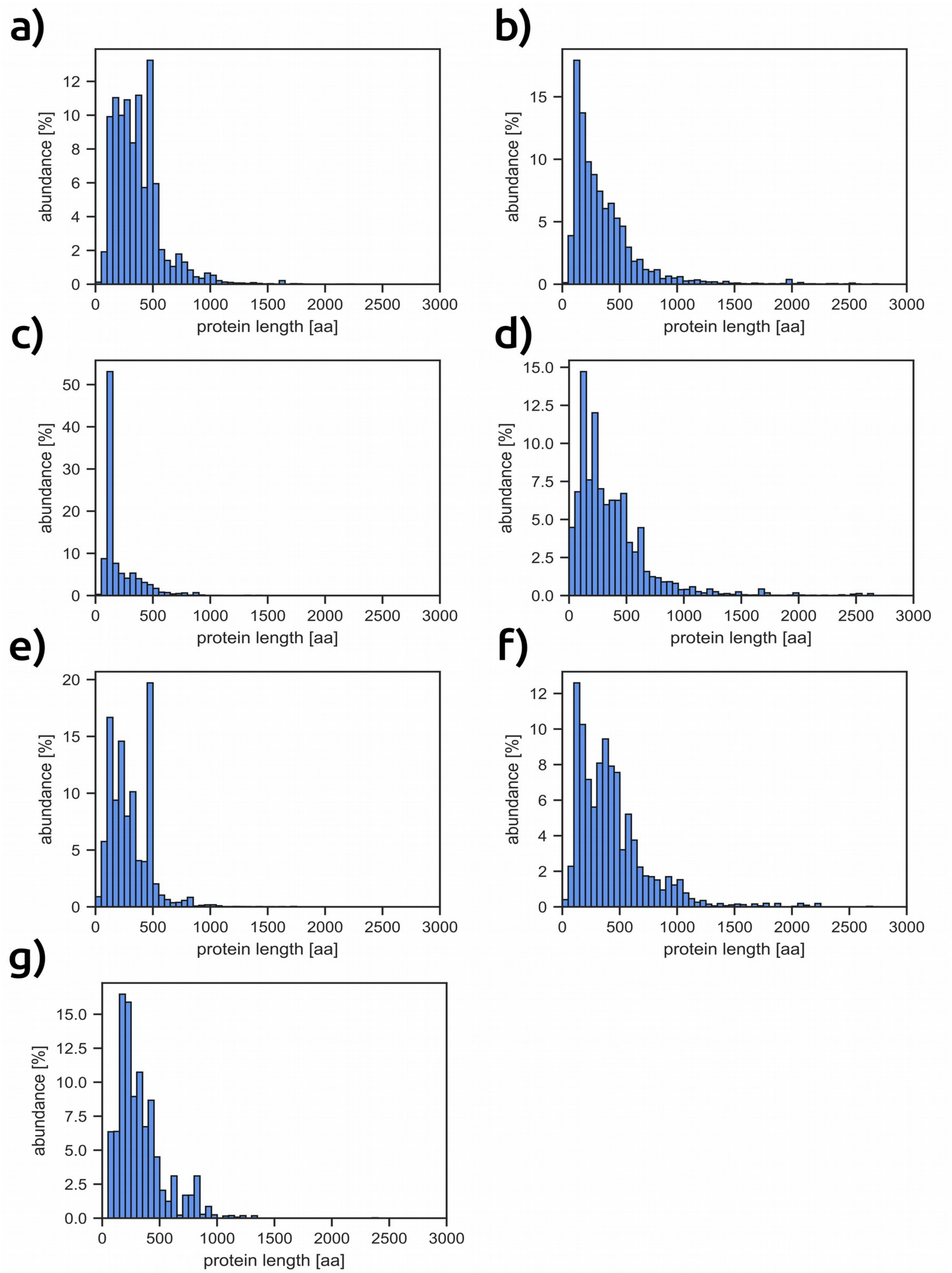

Figure S6 Abundance weighted protein length distribution for the *A. thaliana* (a), *D. melanogaster* (b), *E. coli* (c), *H. sapiens* (d), *O. sativa* (e), *S. cerevisiae* (f) and *T. gammatolerans* (g) proteome deposited in PAXdb. Bin size is 100 a.a.

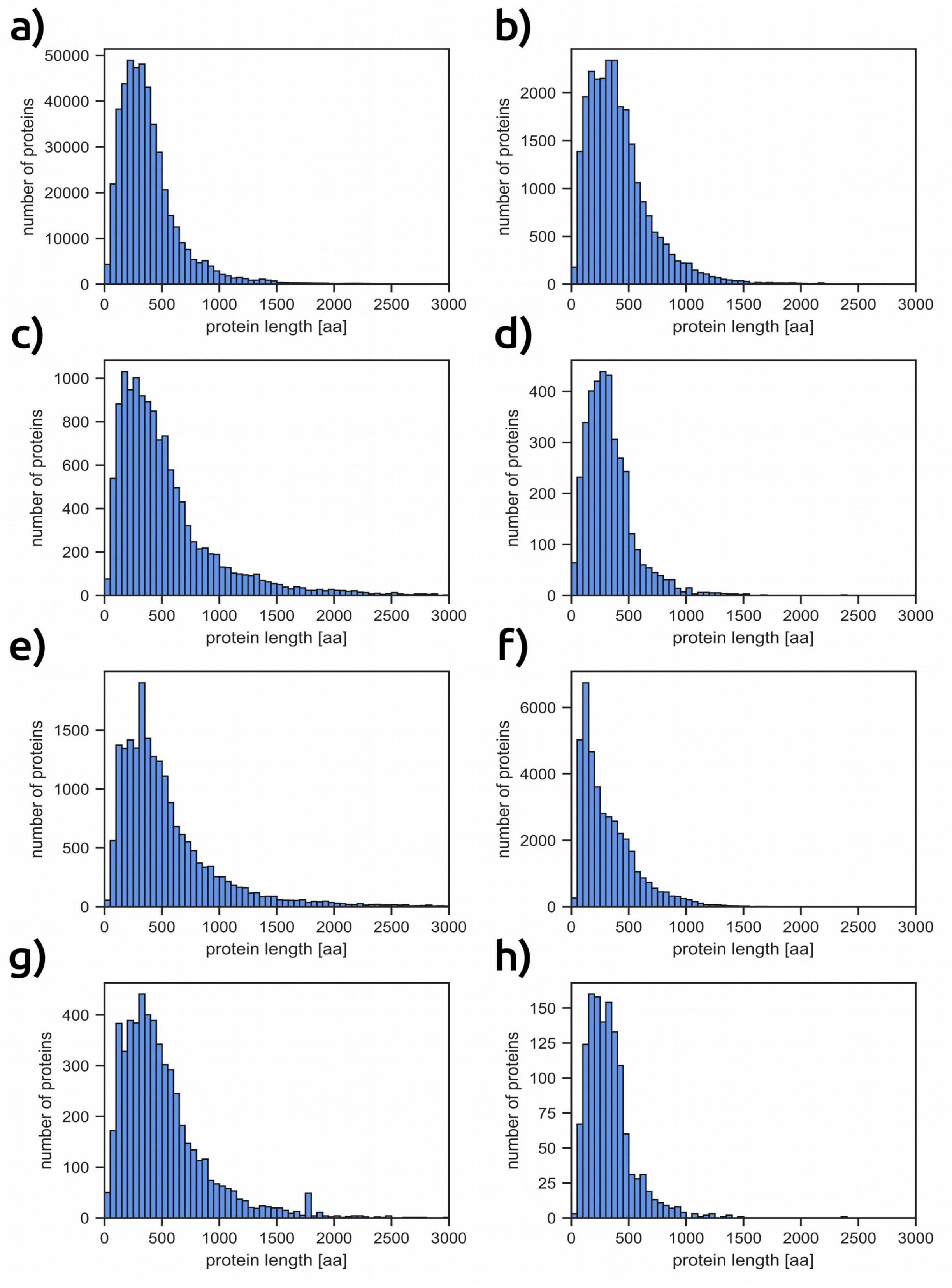

Figure S7 Genomic protein length distribution of cysteine-containing proteins in the SwissProt (a), *A. thaliana* (b), *D. melanogaster* (c), *E. coli* (d), *H. sapiens* (e), *O. sativa* (f), *S. cerevisiae* (g) and *T. gammatolerans* (h) proteome deposited in UniProt. Bin size is 100 a.a.

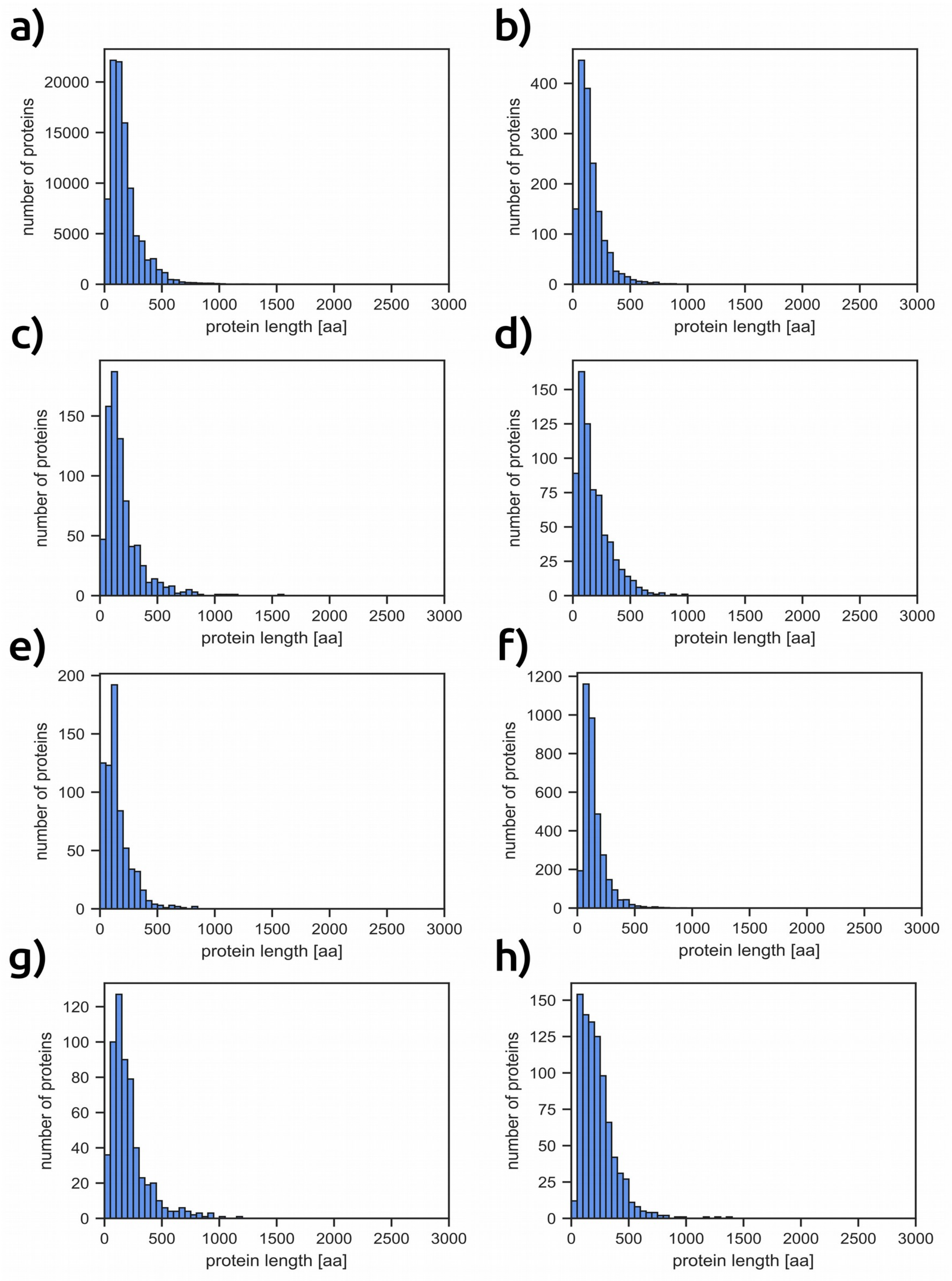

Figure S*8* Genomic protein length distribution of cysteine-free proteins in the SwissProt (a), *A. thaliana* (b), *D. melanogaster* (c), *E. coli* (d), *H. sapiens* (e), *O. sativa* (f), *S. cerevisiae* (g) and *T. gammatolerans* (h) proteome deposited in UniProt. Bin size is 100 a.a.

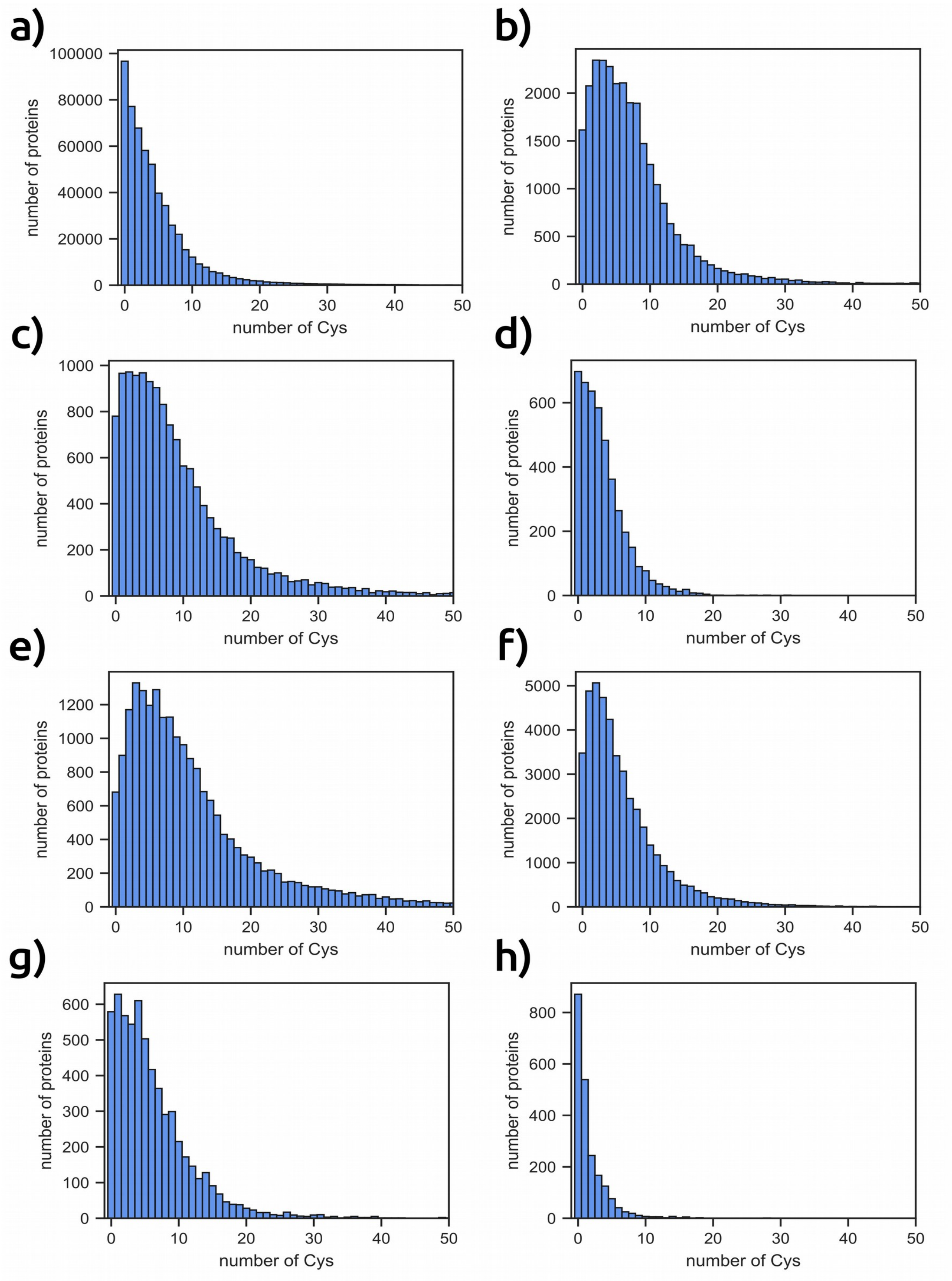

Figure S9 Genomic cysteine distribution in the SwissProt (a), *A. thaliana* (b), *D. melanogaster* (c), *E. coli* (d), *H. sapiens* (e), *O. sativa* (f), *S. cerevisiae* (g) and *T. gammatolerans* (h) proteome deposited in UniProt. Bin size is 1.

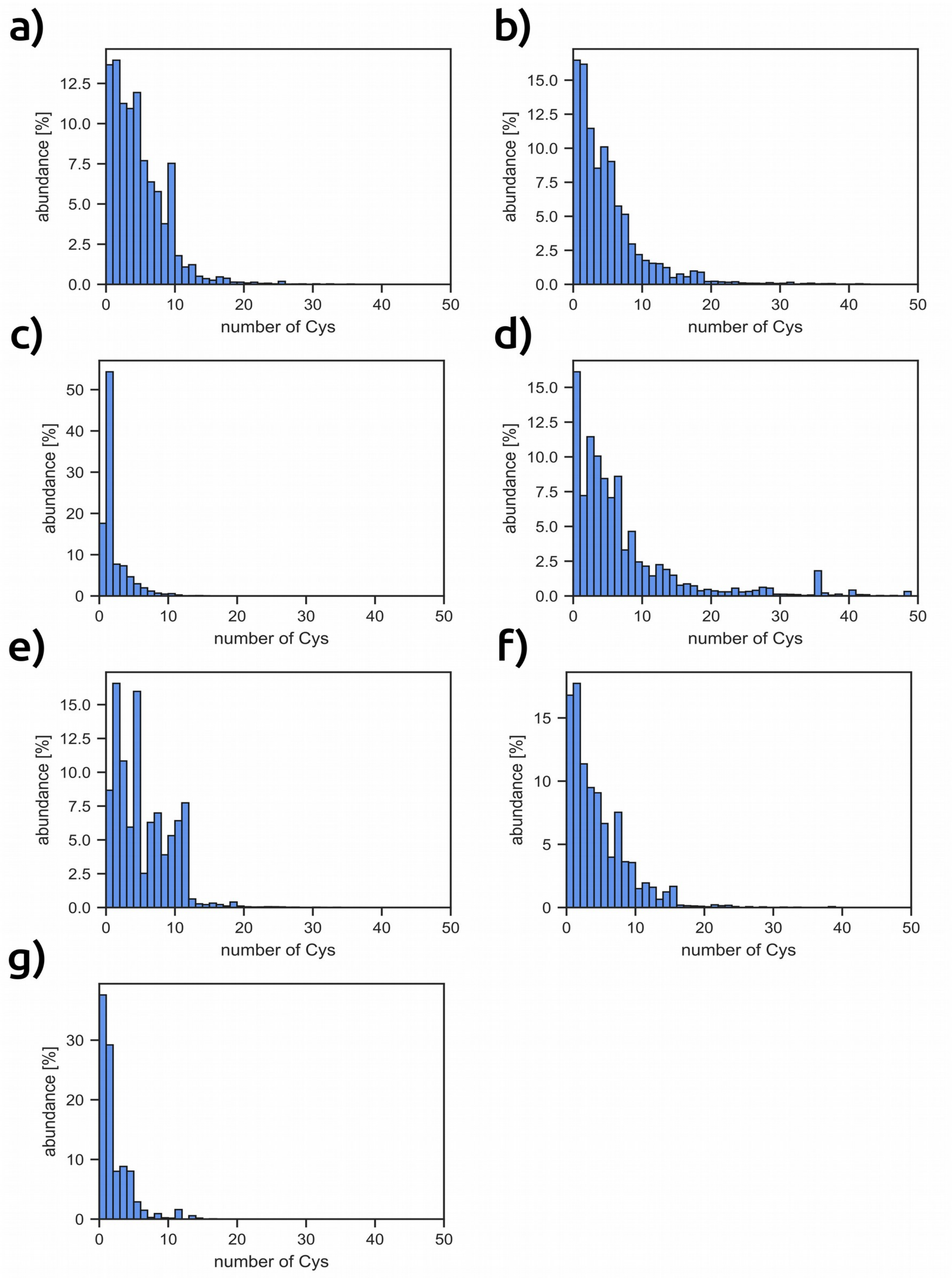

Figure S*10* Abundance weighted cysteine distribution in the *A. thaliana* (a), *D. melanogaster* (b), *E. coli* (c), *H. sapiens* (d), *O. sativa* (e), *S. cerevisiae* (f) and *T. gammatolerans* (g) proteome deposited in PAXdb. Bin size is 1.

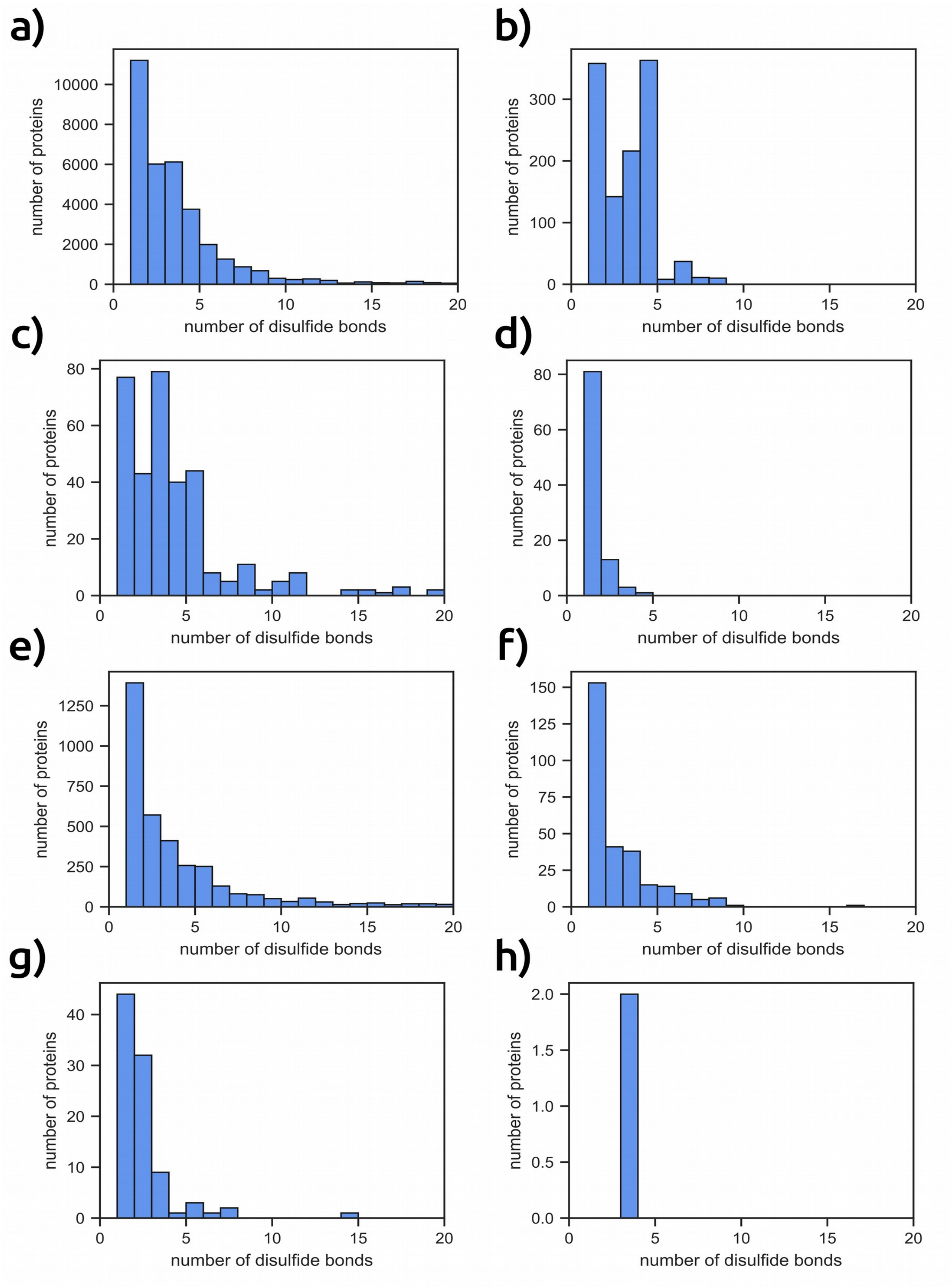

Figure S*11* Genomic disulfide bond distribution of reviewed proteins in the SwissProt (a), *A. thaliana* (b), *D. melanogaster* (c), *E. coli* (d), *H. sapiens* (e), *O. sativa* (f), *S. cerevisiae* (g) and *T. gammatolerans* (h) proteome deposited in UniProt. For *T. gammatolerans* (h) also unreviewed proteins are considered because reviewed proteins contain no disulfide bonds. Bin size is 1.

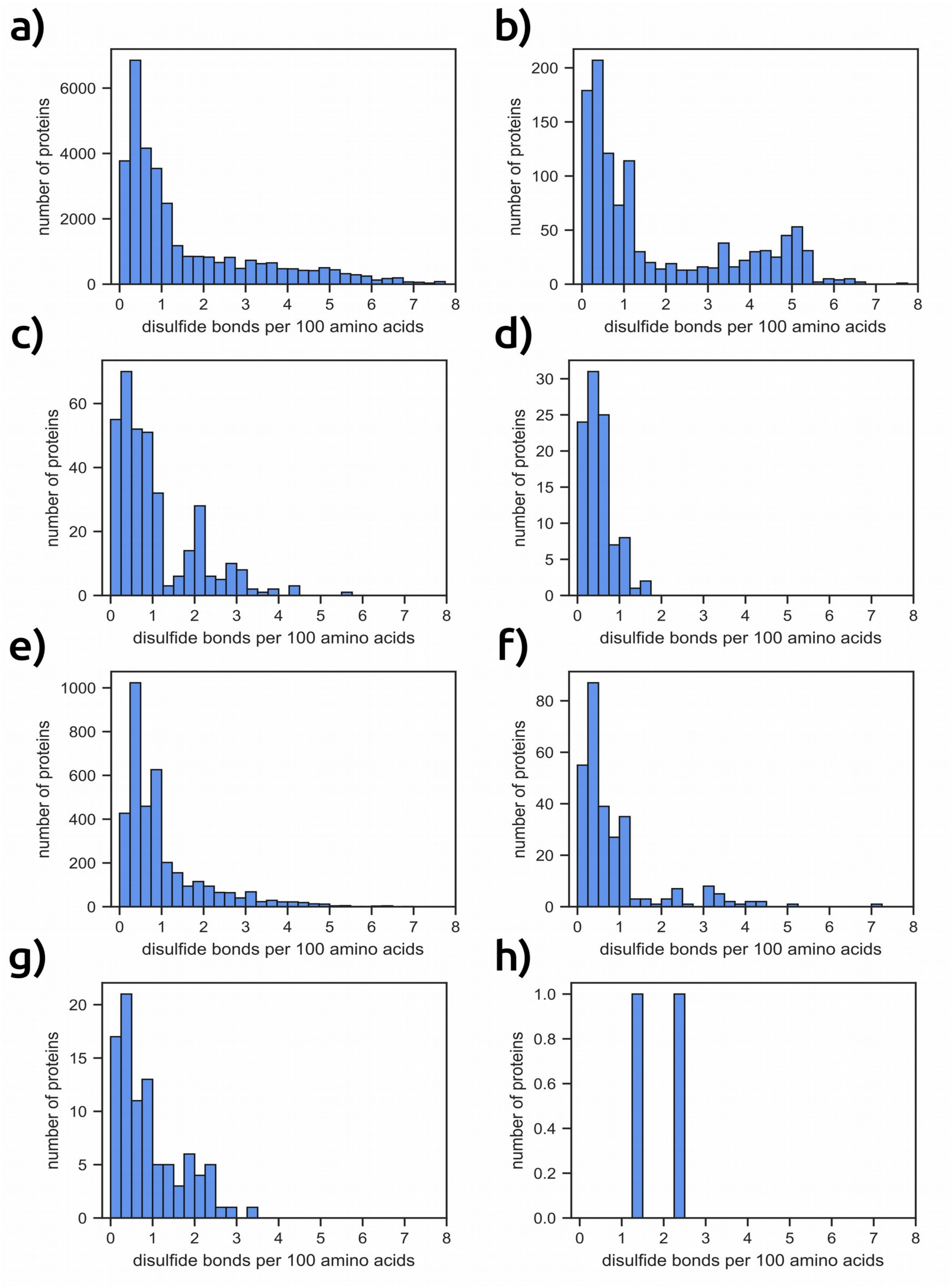

Figure S*12* Disulfide bond frequency distribution of reviewed proteins in the SwissProt (a), *A. thaliana* (b), *D. melanogaster* (c), *E. coli* (d), *H. sapiens* (e), *O. sativa* (f), *S. cerevisiae* (g) and *T. gammatolerans* (h) proteome deposited in UniProt. For *T. gammatolerans* (h) also unreviewed proteins are considered because reviewed proteins contain no disulfide bonds. Bin size is 1.

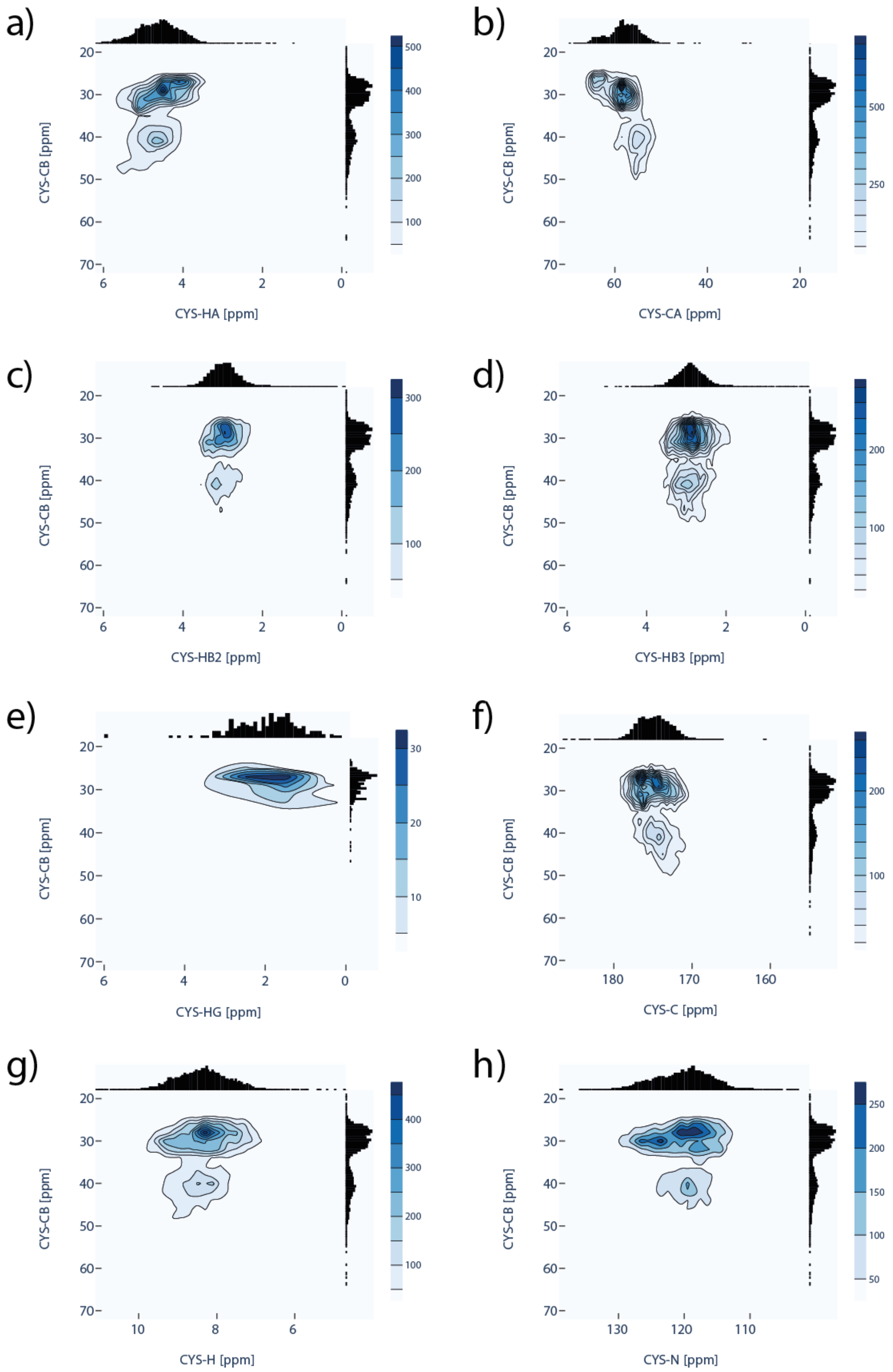

Figure S*13* Chemical shift correlation of cysteine C^{β} and H^{α} (A), C^{α} (B), $H^{\beta 2}$ (C), $H^{\beta 3}$ (D), H^{γ} (E), C' (F), H^{N} (G), N' (H), respectively. Chemical shift data and correlations are obtained and visualized from the Biological Magnetic Resonance Data Bank(BMRB) with the help of a modified PyBMRB python module. Distribution values which are outside ten times the standard deviation were removed from each correlation data set. Contour levels reflect the total number of correlations within.

Supplementary Material

Table S*1*. Proteomic abundance weighted analysis

Species	Number of proteins / proteins with Cys in fasta file	Median length all / proteins with Cys / proteins without Cys	Proteins / proteins with 0 ppm in abundance file	Abundance weighted median length all proteins / proteins with Cys / proteins without Cys	Median Cys per protein (abundance weighted) / proteins with Cys
A.thaliana	27 416 / 25 760 (94%)	348 / 362 / 122	20 185 / 152	337 / 360 / 187	6 (4) / 6 (4)
D.melanogaster	13 937 / 13 110 (94%)	393 / 411 / 144	13 264 / 1	271 / 321 / 150	7 (3) / 7 (4)
E.coli	4 146 / 3 515 (85%)	282 / 301 / 150	4 096 / 367	144 / 144 / 130	3 (1) / 3 (1)
H.sapiens	20 457 / 19 797 (97%)	410 / 421 / 126	19 949 / 611	283 / 338 / 102	9 (4) / 9 (5)
O.sativa	57 939 / 54 152 (93%)	328 / 353 / 105	5 656 / 0	260 / 284 / 145	6 (4) / 6 (4)
S.cerevisiae	6 692 / 6 065 (91%)	359 / 386 / 154	6 440 / 2	363 / 412 / 152	4 (3) / 5 (4)
T.gammatolerans	2 156 / 1 285 (60%)	251 / 298 / 198	1 341 / 0	268 / 335 / 226	1 (1) / 2 (2)
